# Associations Between Neuropathy, Nephropathy and Hearing Loss in Individuals with Type 2 Diabetes

**DOI:** 10.3390/biomedicines14051153

**Published:** 2026-05-20

**Authors:** Joutiar Razay, Jesper Hvass Schmidt, Mette K. Andersen, Jens S. Nielsen, Michael Hecht Olsen, Thomas Bastholm Olesen

**Affiliations:** 1 Steno Diabetes Center Odense, Odense University Hospital, 5000 Odense, Denmark; jens.steen.nielsen@rsyd.dk (J.S.N.); thomas.bastholm.olesen@rsyd.dk (T.B.O.); 2 Research Unit for ORL—Head & Neck Surgery and Audiology, Odense University Hospital, 5000 Odense, Denmark; jesper.schmidt@rsyd.dk; 3 Department of Clinical Research, Faculty of Health Sciences, University of Southern Denmark, 5000 Odense, Denmark; 4 Novo Nordisk Foundation Center for Basic Metabolic Research, University of Copenhagen, 2200 Copenhagen, Denmark; metteandersen@sund.ku.dk; 5 Department of Internal Medicine and Steno Diabetes Center Zealand, Holbæk Hospital, 4300 Holbæk, Denmark; michael.olsen@dadlnet.dk; 6 Department of Clinical Medicine, University of Copenhagen, 2200 Copenhagen, Denmark

**Keywords:** hearing loss, Type 2 diabetes mellitus, neuropathy, nephropathy, chronic low-grade inflammation, observational, cohort study

## Abstract

**Aims**: The aim of this study was to investigate the associations between symptomatic hearing loss (HL), neuropathy, and nephropathy in subjects with Type 2 diabetes mellitus (T2DM). Furthermore, the study evaluated whether HL was associated with chronic low-grade inflammation, assessed based on plasma levels of tumour necrosis factor-alpha (TNF-α), interleukin-6 (IL-6), and high-sensitivity C-reactive protein (hsCRP), and explored potential sex-specific differences. **Materials and Methods**: We included 4245 subjects with T2DM from The Danish Centre for Strategic Research in Type 2 Diabetes cohort. Symptomatic HL was defined using ICD-10 codes. In 2016, a questionnaire was sent out to evaluate neuropathy using the Michigan Neuropathy Screening Instrument (MNSI ≥ 4). Nephropathy was defined as urinary albumin-to-creatinine ratio (UACR) >30 mg/g. Plasma levels of TNF-α, IL-6, and hsCRP were measured at enrolment from 2010 to 2016. Multivariable logistic regression was used, adjusting for covariates. **Results**: Neuropathy was significantly associated with HL (OR = 1.83, 95%CI [1.42, 2.35], *p* < 0.001), and the association was stronger in women (OR = 2.74 [1.81, 4.14], *p* < 0.001) compared to men (OR = 1.44 [1.04, 1.99], *p* < 0.05) (P-interaction = 0.020). No significant association was found between nephropathy and HL. Among inflammatory markers, only the highest tertile of TNF-α levels was significantly associated with HL compared to the lowest tertile (OR = 1.40 [1.07, 1.82], *p* < 0.05) without any sex interaction. **Conclusions**: In subjects with T2DM, neuropathy was associated with symptomatic HL, and the association seemed to be stronger in females. Among chronic low-grade inflammation markers, only TNF-α was significantly associated with symptomatic HL. Additionally, no significant association was found between nephropathy and HL.

## 1. Introduction

According to the World Health Organisation, 6% of the global population suffers from disabling hearing loss (HL), making it the third leading cause of disability worldwide [1,2]. This prevalence is expected to rise further both due to the rapidly ageing population and due to the increasing prevalence of individuals with type 2 diabetes mellitus (T2DM) [3], who have up to twice the risk of developing HL compared to those without diabetes [4,5,6]. One third of individuals living with T2DM have some degree of HL upon audiometric screening [7]. HL in individuals with T2DM has garnered significant interest, as evidence points to a potential link between angiopathy and/or neuropathy and HL [8]. However, findings across studies have been inconsistent, underscoring the complex interplay of pathophysiological mechanisms. Some researchers propose that HL may be classified as a microvascular complication of T2DM similar to diabetic retinopathy [9,10,11], nephropathy [11,12], and neuropathy [13,14]. The precise aetiology of HL in T2DM remains unclear, raising key questions about whether it is primarily driven by microvascular damage to the small arteries of cochlea or by diabetes-related neuropathy affecting the auditory nerve. If microangiopathy is the primary mechanism, HL may co-occur with nephropathy. Conversely, if neuropathy plays a larger role, HL may align more closely with diabetic neuropathy and manifest in patients with peripheral nervous system damage.

The difference in sex plays a significant role in the prevalence and progression of HL. Males are shown to face earlier HL compared to females, potentially due to noise exposure and hormonal influences [15,16]. Conditions such as diabetes mellitus and CVD also contribute to HL, possibly through chronic low-grade inflammation and perhaps with sex-specific differences. Patients living with T2DM often exhibit chronic low-grade inflammation characterised by elevated levels of inflammatory markers such as interleukin-6 (IL-6), high-sensitivity C-reactive protein (hsCRP) [17], and/or tumour necrosis factor-alpha (TNF-α) [18,19]. These markers are implicated in metabolic dysregulation and vascular dysfunction and may directly affect the delicate microstructures of the ear, including its vascularisation and auditory function. Emerging evidence suggests that chronic low-grade inflammation could play a crucial role in HL by impacting both vascular and neural pathways [20].

This study aims to investigate the association between the presence of microvascular complications such as diabetic polyneuropathy (DPN) (assessed using the Michigan Neuropathy Screening Instrument (MNSI) questionnaire) or nephropathy (assessed using the urinary albumin-to-creatinine ratio (UACR)) and the prevalence of HL, defined as having a clinical diagnosis of HL. Furthermore, the study seeks to evaluate whether low-grade chronic inflammation, assessed as plasma levels of TNF-α, IL-6, and hsCRP, is independently associated with HL, and to explore potential sex-specific differences.

## 2. Materials and Methods

Setting: The Danish Centre for Strategic Research in Type 2 Diabetes (DD2) cohort is a nationwide cohort of subjects with recently diagnosed T2DM enrolled from either hospital specialist outpatient clinics or general practitioners’ offices across Denmark since November 2010. At enrolment, each patient underwent an interview, clinical examination, and blood and urine sampling for the DD2 biobank. The unique civil registration number assigned to all Danish citizens allows linkage to Danish health registries, including the Danish National Patient Registry (hospital contacts), the Danish National Health Service Prescription Database (prescriptions), the Danish Civil Registration System (vital status and migration data), and the Danish Diabetes Database for Adults (DDDA) [21]. A detailed overview of registries and study variables is provided in Supplementary Table S1.

Study Population: On June 2016 (hereafter referred to as the “index date”), a neuropathy and pain questionnaire was sent to all 6726 (100%) living DD2 participants enrolled from November 2010 to February 2016, with a median of 2.8 years (interquartile range (IQR) 1.8–3.7) post-enrolment. The questionnaire included MNSI along with questions on anthropometric data and lifestyle factors [22]. A total of 5259 participants returned a valid questionnaire (78.2%). Among these, 4406 had a UACR measurement available (65.5%), and 4245 additionally had inflammatory biomarker data from blood samples collected at enrolment. The present study therefore included 4245 participants (63.1%) with self-reported polyneuropathy data, UACR measured within one year of the questionnaire, and available inflammatory biomarker measurements. The timing of the measurements of UACR, inflammatory biomarkers, and HL diagnoses relative to the MNSI are visualised in Supplementary Figure S1 and specified numerically in Supplementary Figure S2.

Exposures: The study’s primary exposures were as follows:

- Diabetic polyneuropathy (DPN): DPN was assessed using the MNSI and defined as a score ≥4 (sensitivity 40%, specificity 92%) [23].
- Nephropathy: Nephropathy was assessed based on a single UACR measurement from the Danish laboratory database and DDDA and defined as microalbuminuria (30–300 mg/g) or macroalbuminuria (>300 mg/g).
- Chronic low-grade inflammation: Inflammatory markers were measured from blood samples collected at DD2 enrolment (median 2.8 years before the index date). Plasma levels of TNF-α and IL-6 (pg/mL) were quantified using Meso Scale Discovery V-plex immunoassays (Meso Scale Diagnostics, Rockville, MD, USA), with intra- and interplate coefficients of variation of 5% and 14.4% for TNF-α, and 6.1% and 12.3% for IL-6, respectively. Serum hsCRP (mg/L) was measured using an enzyme-linked immunosorbent assay with a time-resolved immuno-fluorometric technique, achieving intra- and inter-assay variation of <5% and <6%, respectively. For the analyses, biomarker levels were categorised into the following tertiles: low, intermediate, and high [24].

Outcome: The primary outcome was a composite endpoint of HL, identified based on ICD-10 codes recorded up to the date of neuropathy questionnaire administration (index date). The codes included noise-induced HL (DH833, DH833A), diseases of the inner ear (DH838, DH839), conductive and sensorineural HL (DH90, DH900), ear disorders associated with diseases classified elsewhere (DH94, DH948), sensorineural HL (DH903, DH904, DH905, DH905A, DH905B, DH905C, DH905D, DH905E, DH906, DH907, DH908), and other diagnoses of HL (DH91, DH911, DH912, DH912A, DH913, DH918, DH918A, DH919, DH919A, DH919B). The different event numbers are shown in Supplementary Table S2.

Covariates: Potential confounders and mediators were extracted from the DD2 cohort questionnaire, neuropathy questionnaire (BMI and smoking status), and health registries:

- Confounders: age, sex, BMI, smoking status, alcohol consumption and polygenic risk score (PRS) for HL.
- Mediators: low-density lipoprotein cholesterol (LDL-C), HbA1c, medication use (glucose-, lipid-, or blood pressure-lowering medication) within 1 year of the index date and previous cardiovascular disease (CVD).

Genotyping was performed with the Global Screening Array-24 v2.0 or v3.0 chip (Illumina, San Diego, CA, USA), and genotypes were called using the GenCall algorithm in GenomeStudio software (version 2.0, Illumina, San Diego, CA, USA). We performed standard quality control and imputation, with the Haplotype Reference Consortium data as a reference panel. A PRS for HL summarising genetic risk by combining the small effects of many common genetic variants into a single score was calculated and included to adjust for inherited susceptibility to HL [25].

Missing Data: Missing covariate data were handled using multiple imputation with chained equations. Missingness was highest for LDL cholesterol (*n* = 1608; 37.9%), followed by the PRS for HL (*n* = 320; 7.0%), BMI (*n* = 75; 1.8%), and HbA1c (*n* = 25; 0.6%). The imputation model included all covariates and exposures, and the outcome. Twenty imputed datasets were generated and combined using Rubin’s rules. The distributions of observed and imputed values were visually compared, including inspection of the first and twentieth imputed datasets, and showed good agreement (Supplementary Figure S3).

Statistical Analyses: Descriptive statistics are presented as medians with interquartile ranges (IQR) for continuous variables and proportions (*n* [%]) for categorical variables, stratified by the presence or absence of an HL diagnosis on the index date. Comparisons between groups were made using the Mann–Whitney U test for continuous variables and Pearson’s chi-square test for categorical variables. Missing covariate data were treated using multiple imputation with chained equations to maximise precision and reduce selection bias, as previously described [26].

Logistic regression models examining association of the diagnosis of HL with MNSI, nephropathy (UACR), and markers of low-grade chronic inflammation (TNF-α, hsCRP, IL-6), presented as odds ratios (OR), 95% confidence intervals (CI) in brackets, and *p*-values, for the following three hierarchical models:

- Model 1: Adjusted for age and sex.
- Model 2: Further adjusted for HbA1c; LDL-C; BMI; smoking status; previous cardiovascular disease; and glucose-, lipid-, and blood pressure-lowering treatment.
- Model 3: Further adjusted for PRS for HL and for inflammatory markers (TNF-α, IL-6, and hsCRP) in analyses of neuropathy or adjusted for neuropathy and nephropathy in analyses of chronic low-grade inflammation.

To explore whether the association between exposures and prevalent HL was modified by age or sex, interaction terms for age and sex were included in the logistic regression models.

All statistical tests were two-sided, and a *p*-value below 0.05 was considered statistically significant. Analyses were conducted using Stata version 18 (StataCorp LLC, College Station, TX, USA).

Research Ethics and Informed Consent: The DD2 study received approval from the Danish National Committee on Health Research Ethics (S-20100082) and the Danish Data Protection Agency (2008-58-0035). All participants provided written informed consent.

## 3. Results

### 3.1. Baseline Characteristics

Table 1 shows the differences in baseline characteristics between participants with and without diagnosed HL. Participants with symptomatic HL were significantly older (71.0 years vs. 64.7 years, *p* < 0.001) and more frequently male (71.2% vs. 57.3%, *p* < 0.001). Participants with HL had a higher prevalence of previous cardiovascular disease (39.8% vs. 26.4%, *p* < 0.001) and neuropathy (24.5% vs. 16.8%, *p* < 0.001), lower eGFR (82.2 vs. 91.5 mL min^−1^ (1.73 m^2^)^−1^, *p* < 0.001), more albuminuria (26.3% vs. 18.9%, *p* < 0.01), and slightly higher levels of TNF-α (1.04 vs. 0.95 pg/mL, *p* < 0.001) and of IL-6 (1.25 vs. 1.17 pg/mL, *p* < 0.01) compared to those without. Among the 445 participants with a registered HL diagnosis, 144 (32.4%) had the HL diagnosis recorded more than 10 years before DD2 enrolment, 210 (47.2%) had the diagnosis recorded within the 10 years preceding DD2 enrolment, and 91 (20.4%) were diagnosed between DD2 enrolment (measurements of biomarkers) and the index date (shown in Supplementary Figure S2).

### 3.2. Regression Results

**Figure 1:**
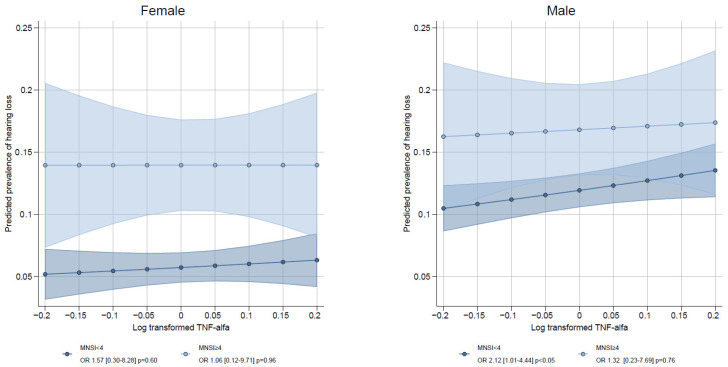
Predicted prevalence of symptomatic hearing loss according to log-transformed TNF-α, stratified by sex and neuropathy status (MNSI < 4 vs. ≥ 4). The interaction between TNF- α and neuropathy was not significant.

Table 2 and Table 3 show the results of logistic regression models, exploring associations between chronic low-grade inflammation, neuropathy (assessed by questionnaire), and nephropathy (assessed by UACR) and HL. A significant association between neuropathy and symptomatic HL was found in all three models, and in model 3 (OR = 1.83, 95%CI [1.42, 2.35], *p* < 0.001). In the sex-specific analyses, the associations were still significant for both sexes, but with a significantly stronger association in females (OR = 2.74 [1.81, 4.14], *p* < 0.001) compared to males (OR 1.44 [1.04, 1.99], *p* < 0.05) (P-interaction = 0.020) (Table 2). Predicted prevalences according to sex and neuropathy status were as follows: among women, the predicted prevalence of HL was 5.8% (95% CI 4.6–6.9) in those without neuropathy and 13.9% (95% CI 10.3–17.6) in those with neuropathy. Among men, the corresponding prevalences were 11.9% (95% CI 10.6–13.3) without neuropathy and 16.9% (95% CI 13.2–20.5) with neuropathy (Supplementary Table S3). Among inflammatory markers, only the highest tertile of TNF-α levels was significantly associated with symptomatic HL compared to the lowest tertile (OR = 1.40 [1.07, 1.82], *p* < 0.05) without any sex interaction across all three models (Table 3). Estimated prevalence of HL was evaluated in sex-stratified analyses with TNF-α included as a continuous log-transformed variable (Figure 1). In men without neuropathy, higher TNF-α levels were associated with a higher prevalence of HL (OR 2.12 [1.01–4.44]; *p* < 0.05), but not in men with neuropathy (OR 1.32 [0.23–7.69]; *p* = 0.76), without significant interaction (*p* = 0.57). Among women, TNF-α was not associated with HL regardless of neuropathy status. (Figure 1). Symptomatic HL was not significantly associated with micro- nor macroalbuminuria in any of the models. We did not find any significant sex interaction for the associations between UACR and HL or between inflammatory markers and HL.

## 4. Discussion

Our study had three primary findings: (1) Neuropathy was significantly associated with symptomatic HL, possibly with a stronger association observed in females compared to males. (2) Nephropathy was not significantly associated with symptomatic HL. Among the inflammatory markers examined, only participants in the highest tertile of TNF-α had higher odds of symptomatic HL, whereas no consistent associations were observed for hsCRP or IL-6.

Regarding neuropathy, our findings were consistent with prior studies highlighting neuropathy as a potential risk factor for symptomatic HL in patients living with T2DM. Previous cross-sectional studies have shown that diabetic neuropathy, also assessed using MNSI, was independently associated with high-frequency HL and that a dose–response relationship existed between neuropathy severity and HL, with the highest prevalence among those with moderate-to-severe neuropathy [13,14]. These findings align with our observation of an association between neuropathy and symptomatic HL across all levels of adjustment. A cross-sectional study explored the relationship between neuropathy and HL in subjects with and without diabetes, identifying neuropathy as a factor for impairments in vision and hearing [27]. Their findings suggest that neuropathy-related HL may transcend diabetes-specific mechanisms, a hypothesis supported by the persistence of our findings after adjustment for metabolic and inflammatory markers. In the present study, the association between neuropathy and symptomatic HL appeared stronger in women than in men. The reasons for this difference are unclear. Although some studies have suggested that men may have a higher lifetime exposure to occupational or recreational noise, which could contribute to HL independent of neuropathy [28,29], we did not have detailed information on noise exposure in the present study. Consequently, the observed sex difference should be interpreted cautiously, as several subgroup and interaction analyses were performed without adjustment for multiple testing, and may thus reflect a combination of biological differences, differences in risk factor profiles, chance findings, or residual confounding.

In our large cohort with subjects living with T2DM, we did not demonstrate a substantial association between symptomatic HL and nephropathy after adjusting for potential confounders. This contrasts with prior studies demonstrating that elevated UACR and declining renal function were associated with HL [11,12,30]. However, these studies used audiometric testing, typically PTA ≥ 25 dB, to detect HL, whereas our study relied on ICD-10 codes for HL, which only capture symptomatic and therefore more severe cases of HL. This difference in HL definition may explain the discrepancy, as milder and probably earlier HL, potentially linked to nephropathy, might not have been identified in our study. These findings underscore the importance of distinguishing between subclinical and clinical (symptomatic) HL in future research. Incorporating audiometric data may help clarify the role of nephropathy and microvascular changes in the mechanisms underlying HL. Our findings of an independent association between symptomatic HL and TNF-α align with prior studies that highlight the potential harmful effects of TNF-α on auditory function [18]. An experimental animal study demonstrated that acute TNF-α exposure induces damage to the cochlear synapse, reducing auditory nerve activity without affecting hearing thresholds or hair cell integrity [31]. These results may suggest a possible neurotoxic effect of TNF-α on auditory synapses, which may mirror the mechanisms underlying HL observed in our study. However, the present findings should be interpreted cautiously. The association was modest in magnitude, limited to the highest TNF-α tertile, and was in contrast to previous studies [17,19,32] not accompanied by similar associations for hsCRP or IL-6. Moreover, although the overall association with TNF-α remained after multivariable adjustment, it was not consistently observed across sex-stratified analyses. Thus, our data do not support a broad or consistent association between systemic chronic low-grade inflammation and symptomatic HL in individuals with T2DM. Again, a key difference between our study and the current literature lies in definition and measurement of HL. Prior studies have often used audiometry, detecting milder and even asymptomatic cases of HL, whereas we used ICD-10 codes to define HL, detecting only symptomatic cases. The fact that we only detected symptomatic and often later HL may explain the missing associations between HL and early inflammatory markers such as hsCRP and IL-6 that are often associated with early microvascular changes such as elevated UACR [33,34,35]. Previous studies have shown that PRS for HL is associated with increased self-reported HL and slightly elevated pure-tone audiometric thresholds [25,36]. In the present study, the associations between both neuropathy and circulating TNF-α and symptomatic HL remained statistically significant after adjustment for the PRS for HL, indicating that these associations are largely independent of inherited genetic susceptibility. The lack of association between IL-6, hsCRP and HL does not support a major role of low-grade inflammation for the presence of symptomatic HL among individuals with T2DM. However, the modest association observed with the highest tertile of TNF-α, known to be produced by macrophages but also by microglia and neurons, may suggest a role for neuroinflammation in development of HL in T2DM [37,38].

### Strengths and Limitations

This observational, cross-sectional study cannot establish causality, and residual confounding cannot be fully excluded despite consistent associations across multiple adjusted models. Neuropathy was assessed using the self-reported MNSI questionnaire, which has a reported sensitivity of 40% and specificity of 92%. This may introduce recall bias or subjective reporting, and the lack of a clinical examination to complement the MNSI is a limitation, as it may have resulted in some misclassification, particularly among individuals with mild or asymptomatic neuropathy. However, any misclassification would likely have weakened the observed associations, meaning the true relationship between symptomatic HL and neuropathy may be even stronger than reported.

As our study defined HL through ICD-10 codes rather than audiometric testing, the findings are not directly comparable to PTA-based studies, which capture a wider range of hearing impairment severity, including subclinical cases. Only individuals with symptoms of HL have sought medical attention and thereafter received a confirmative diagnosis, potentially underrepresenting mild or asymptomatic cases of HL. However, our findings remain clinically relevant, as they focus on individuals with more advanced cases of HL. Additionally, patients with more diabetes-related complications, specifically symptomatic neuropathy, tend to visit healthcare services more frequently, which may increase their likelihood of receiving an HL diagnosis and thereby introduce detection bias. Nephropathy, which is asymptomatic, may not lead to the same degree of detection bias. To address potential detection bias, we examined the timing of HL diagnoses relative to DD2 enrolment. When we grouped participants by timing of HL diagnosis, the association with neuropathy was similar across all three groups (Supplementary Table S4). This suggested that the association was unlikely to be explained by the fact that patients with neuropathy may have more healthcare contacts (detection bias). This was further supported by Supplementary Table S5, demonstrating that the incidence rate for the 91 HL diagnosis given after enrolment in DD2 remained relatively stable across follow-up intervals rather than clustering close to the index date.

The study population consisted of subjects living with T2DM in Denmark, which may limit generalisability to other populations. Our study lacked access to occupational history and noise exposure data, which are recognised risk factors for HL. Because occupational noise exposure is typically lower among women, residual confounding by noise may be less pronounced in this group, which could contribute to the larger observed effect estimates [28].

HL diagnoses often preceded the assessment of neuropathy, nephropathy, and inflammatory biomarkers by several years. This temporal separation may have resulted in exposure misclassification, which would primarily be expected to attenuate true associations rather than generate spurious findings. Consequently, null associations (particularly for nephropathy and inflammatory markers) should be interpreted with caution. However, both diabetic neuropathy and chronic low-grade inflammation are well-established slowly progressive conditions that are likely to have been present for a considerable period prior to clinical detection. Prospective studies with repeated measurements are needed to fully establish the temporal relationships between these conditions.

Finally, several prespecified subgroup and interaction analyses were performed. Multiple comparisons increase the possibility that some findings occurred by chance; the observed sex interaction for neuropathy should therefore be interpreted cautiously and considered hypothesis-generating.

Despite these limitations, this study benefits from a large, well-characterised cohort with linkage to national health registries. The use of validated tools for neuropathy assessment, combined with comprehensive biomarker analysis and sex-stratified analysis, strengthens the validity and clinical relevance of our findings.

## 5. Conclusions

In conclusion, our study demonstrated a significant and independent association between neuropathy and symptomatic HL in subjects with T2DM. Exploratory analyses suggested possible sex differences in this association. Although TNF-α was associated with symptomatic HL, the lack of consistent associations across inflammatory markers does not support a broad association between markers of systemic low-grade inflammation and symptomatic HL.

## Figures and Tables

**Table 1:** Baseline characteristics—patients with symptomatic hearing loss compared to patients without.

Columns by Category		Without Symptomatic Hearing Loss	With Symptomatic Hearing Loss	Total	*p*-Value
N, *n* (%)		3800 (89.5)	445 (10.5)	4245 (100)	
Age, median (IQR)		64.7 (56.0–70.9)	71.0 (66.5–77.0)	65.6 (56.7–71.6)	<0.001
Sex, *n* (%)	Female	1622 (42.7)	128 (28.8)	1750 (41.2)	<0.001
BMI, median (IQR)		29.7 (26.4–33.5)	29.4 (26.2–32.8)	29.6 (26.4–33.5)	0.27
Smoking, *n* (%)					
	Never	1351 (35.6)	138 (31.0)	1489 (35.1)	<0.001
	Previous smoker	1676 (44.1)	241 (54.2)	1917 (45.2)	
	Current smoker	763 (20.1)	65 (14.6)	828 (19.5)	
HbA1c mmol/L, median (IQR)		49.0 (44.0–55.0)	48.0 (44.0–52.8)	49.0 (44.0–55.0)	<0.001
Total cholesterol mmol/L, median (IQR)		4.1 (3.6–4.7)	4.0 (3.4–4.7)	4.1 (3.6–4.7)	0.085
LDL-C mmol/L, median (IQR)		1.9 (1.5–2.5)	1.9 (1.4–2.5)	1.9 (1.5–2.5)	0.47
HDL-C mmol/L, median (IQR)		1.2 (1.0–1.5)	1.2 (1.0–1.5)	1.2 (1.0–1.5)	0.83
TG mmol/L, median (IQR)		1.7 (1.2–2.5)	1.7 (1.1–2.4)	1.7 (1.2–2.4)	0.24
Previous CVD, *n* (%)	Yes	1002 (26.4)	177 (39.8)	1179 (27.8)	<0.001
Antihypertensive medication, *n* (%)					
	1–2	1765 (46.4)	208 (46.7)	1973 (46.5)	
	≥3	750 (19.7)	119 (26.7)	869 (20.5)	<0.001
Loop diuretic use, *n* (%)	≥1	377 (9.9)	75 (16.9)	452 (10.6)	<0.001
Aspirin use, *n* (%)	≥1	985 (25.9)	157 (35.3)	1142 (26.9)	<0.001
DM medication 1 year prior to study, *n* (%)					
	None	444 (11.7)	77 (17.3)	521 (12.3)	<0.01
	Non-insulin treatment	2977 (78.3)	330 (74.2)	3307 (77.9)	
	Insulin with or without non-insulin treatment	379 (10.0)	38 (8.5)	417 (9.8)	
Lipid-lowering medication, *n* (%)	≥1	3009 (79.2)	357 (80.2)	3366 (79.3)	0.61
UACR, *n* (%)					
	<30	3080 (81.1)	328 (73.7)	3408 (80.3)	<0.01
	30–300	634 (16.7)	102 (22.9)	736 (17.3)	
	>300	86 (2.3)	15 (3.4)	101 (2.4)	
eGFR, median (IQR)		91.5 (76.5–100.8)	82.2 (66.2–93.2)	90.5 (75.1–100.0)	<0.001
MNSI, *n* (%)	≥4	639 (16.8)	109 (24.5)	748 (17.6)	<0.001
hsCRP mg/mL, median (IQR)		1.86 (0.81–4.09)	1.80 (0.74–3.71)	1.85 (0.81–4.03)	0.083
TNF-α pg/mL, median (IQR)		0.95 (0.78–1.15)	1.04 (0.84–1.25)	0.95 (0.79–1.17)	<0.001
IL-6 pg/mL, median (IQR)		1.17 (0.80–1.79)	1.25 (0.87–1.87)	1.18 (0.81–1.80)	<0.01
PRS for HL, median (IQR)		0.28 (−0.14–0.71)	0.27 (−0.1–0.75)	0.28 (−0.13–0.72)	0.21

**Table 2:** Associations between neuropathy and nephropathy (assessed as MNSI and UACR) and symptomatic hearing loss.

Overall, N = 4245									
	Model 1			Model 2			Model 3		
	OR	95% CI	*p*-Value	OR	95% CI	*p*-Value	OR	95% CI	*p*-Value
Neuropathy									
MNSI ≥ 4	1.90	[1.49, 2.43]	<0.001	1.86	[1.44, 2.39]	<0.001	1.83	[1.42, 2.35]	<0.001
Nephropathy									
UACR 30–300 mg/g	1.22	[0.95, 1.57]	0.12	1.24	[0.96, 1.60]	0.10	1.20	[0.92, 1.55]	0.17
UACR > 300 mg/g	1.26	[0.70, 2.26]	0.44	1.22	[0.67, 2.21]	0.52	1.12	[0.61, 2.05]	0.71
**Male, N = 2495**									
	**Model 1**			**Model 2**			**Model 3**		
	**OR**	**95% CI**	***p*-Value**	**OR**	**95% CI**	***p*-value**	**OR**	**95% CI**	***p*-Value**
Neuropathy									
MNSI ≥ 4	1.54	[1.12, 2.10]	<0.01	1.46	[1.05, 2.01]	<0.05	1.44	[1.04, 1.99]	<0.05
Nephropathy									
UACR 30–300 mg/g	1.25	[0.93, 1.66]	0.14	1.26	[0.94, 1.70]	0.13	1.21	[0.89, 1.64]	0.22
UACR > 300 mg/g	1.44	[0.77, 2.68]	0.25	1.36	[0.72, 2.57]	0.35	1.23	[0.64, 2.35]	0.54
**Female, N = 1750**									
	**Model 1**			**Model 2**			**Model 3**		
	**OR**	**95% CI**	***p*-Value**	**OR**	**95% CI**	***p*-Value**	**OR**	**95% CI**	***p*-Value**
Neuropathy									
MNSI ≥ 4	2.77	[1.87, 4.12]	<0.001	2.77	[1.83, 4.17]	<0.001	2.74	[1.81, 4.14]	<0.001
Nephropathy									
UACR 30–300 mg/g	1.15	[0.70, 1.89]	0.57	1.20	[0.72, 1.99]	0.48	1.17	[0.71, 1.95]	0.54
UACR > 300 mg/g	0.49	[0.064, 3.78]	0.49	0.51	[0.064, 3.97]	0.52	0.50	[0.063, 3.99]	0.52

OR, Odds Ratio; CI, Confidence Interval; MNSI, Michigan Neuropathy Screening Instrument; UACR, Urine Albumin-to-Creatinine Ratio. Model 1: Adjusted for age and sex. Model 2: Further adjusted for HbA1c; LDL-C; BMI; smoking status; previous cardiovascular disease; and glucose-, lipid-, and blood pressure-lowering treatment. Model 3: Further adjusted for PRS for HL and inflammatory markers (TNF-α, IL-6, and hsCRP).

**Table 3:** Associations between chronic low-grade inflammation (assessed as serum levels of hsCRP, IL-6 and TNF-α) and symptomatic hearing loss.

Overall, N = 4245									
	Model 1			Model 2			Model 3		
	OR	95% CI	*p*-Value	OR	95% CI	*p*-Value	OR	95% CI	*p*-Value
3 tertiles of hsCRP									
2	1.09	[0.86, 1.39]	0.48	1.08	[0.84, 1.39]	0.53	1.03	[0.80, 1.33]	0.82
3	1.06	[0.82, 1.37]	0.65	1.01	[0.77, 1.32]	0.94	0.93	[0.69, 1.25]	0.65
3 tertiles of IL-6									
2	1.21	[0.94, 1.56]	0.15	1.19	[0.92, 1.55]	0.19	1.11	[0.85, 1.46]	0.43
3	1.16	[0.90, 1.50]	0.25	1.10	[0.84, 1.44]	0.47	1.01	[0.75, 1.37]	0.95
3 tertiles of TNF-α									
2	1.11	[0.85, 1.45]	0.46	1.09	[0.83, 1.43]	0.53	1.07	[0.82, 1.41]	0.62
3	1.48	[1.15, 1.91]	<0.01	1.45	[1.12, 1.88]	<0.005	1.40	[1.07, 1.82]	<0.05
**Male, N = 2495**									
	**Model 1**			**Model 2**			**Model 3**		
	**OR**	**95% CI**	***p*-Value**	**OR**	**95% CI**	***p*-Value**	**OR**	**95% CI**	***p*-Value**
3 tertiles of hsCRP									
2	1.15	[0.87, 1.53]	0.33	1.14	[0.85, 1.53]	0.38	1.05	[0.78, 1.42]	0.74
3	1.04	[0.77, 1.42]	0.79	0.98	[0.71, 1.36]	0.90	0.82	[0.57, 1.17]	0.27
3 tertiles of IL-6									
2	1.26	[0.93, 1.72]	0.14	1.22	[0.89, 1.68]	0.21	1.21	[0.83, 1.61]	0.39
3	1.32	[0.98, 1.80]	0.07	1.25	[0.91, 1.73]	0.17	1.23	[0.83, 1.73]	0.32
3 tertiles of TNF-α									
2	1.02	[0.74, 1.40]	0.89	1.02	[0.73, 1.40]	0.92	0.99	[0.71, 1.37]	0.94
3	1.49	[1.10, 2.01]	<0.05	1.48	[1.09, 2.02]	<0.05	1.40	[1.01, 1.94]	<0.05
**Female, N = 1750**									
	**Model 1**			**Model 2**			**Model 3**		
	**OR**	**95% CI**	***p*-Value**	**OR**	**95% CI**	***p*-Value**	**OR**	**95% CI**	***p*-Value**
3 tertiles of hsCRP									
2	0.95	[0.59, 1.51]	0.82	0.97	[0.60, 1.56]	0.89	0.96	[0.58, 1.50]	0.86
3	1.07	[0.68, 1.67]	0.78	1.04	[0.64, 1.70]	0.86	1.19	[0.58, 1.54]	0.51
3 tertiles of IL-6									
2	1.09	[0.69, 1.70]	0.72	1.09	[0.69, 1.73]	0.71	1.01	[0.65, 1.66]	0.98
3	0.85	[0.53, 1.36]	0.50	0.78	[0.48, 1.29]	0.34	0.64	[0.43, 1.17]	0.11
3 tertiles of TNF-α									
2	1.35	[0.82, 2.23]	0.24	1.32	[0.79, 2.20]	0.29	1.34	[0.80, 2.25]	0.27
3	1.49	[0.92, 2.41]	0.10	1.45	[0.88, 2.37]	0.14	1.54	[0.92, 2.57]	0.10

OR, Odds Ratio; CI, Confidence Interval; hsCRP, High-Sensitivity C-Reactive Protein; IL-6, Interleukin-6; TNF-α, Tumour Necrosis Factor Alpha. Tertile ranges: TNF-α (T1: <0.84, T2: 0.84–1.08, T3: >1.08); IL-6 (T1: <0.93, T2: 0.93–1.53, T3: >1.53); hsCRP (T1: <1.08, T2: 1.08–3.07, T3: >3.07). Model 1: Adjusted for age and sex. Model 2: Further adjusted for HbA1c; LDL-C; BMI; smoking status; previous cardiovascular disease; and glucose-, lipid-, and blood pressure-lowering treatment. Model 3: Further adjusted for PRS for HL, neuropathy and nephropathy.

## Data Availability

Danish data protection regulations prohibit the public sharing of individual-level patient data. Access to the national health registry data used in this study can, however, be granted to researchers affiliated with authorised institutions upon application to the Danish Health Data Authority (forskerservice@sundhedsdata.dk). Data from the DD2 cohort are available following a separate application process via the DD2 website (https://dd2.dk/forskning/ansoeg-om-data, accessed on 9 February 2026).

## Supplementary Materials

The following supporting information can be downloaded at: https://www.mdpi.com/article/10.3390/biomedicines14051153/s1, Supplementary Table S1: DD2 inclusion criteria, definitions, and codes. Supplementary Table S2: DD2 inclusion criteria, definitions, and codes. Supplementary Table S3: Age- and sex-adjusted association between neuropathy and hearing loss (HL) stratified by timing of HL diagnosis relative to DD2 enrolment. Supplementary Table S4: Incidence of hearing loss diagnoses after DD2 enrolment according to time between enrolment and index date. Supplementary Table S5: Predicted prevalence of hearing loss by sex, age and neuropathy. Supplementary Figure S1: Timing of exposure measurements and hearing loss diagnoses relative to the index date. Supplementary Figure S2: Distribution of observed and imputed values for covariates with missing data. Supplementary Figure S3: Timing of hearing loss diagnoses in relation to DD2 enrolment and index date.

## Abbreviations

The following abbreviations are used in this manuscript:

HL	Hearing loss
T2DM	Type 2 diabetes mellitus
TNF-α	Tumour necrosis factor-alpha
IL-6	Interleukin-6
hsCRP	High-sensitivity C-reactive protein
MNSI	Michigan Neuropathy Screening Instrument
DPN	Diabetic polyneuropathy
UACR	Urine albumin-to-creatinine ratio
DD2	The Danish Centre for Strategic Research in Type 2 Diabetes
DDDA	Danish Diabetes Database for Adults
ICD-10	International Classification of Diseases, 10th revision
BMI	Body Mass Index
HbA1c	Haemoglobin A1c
LDL-C	Low-density lipoprotein cholesterol
HDL-C	High-density lipoprotein cholesterol
TG	Triglycerides
eGFR	Estimated glomerular filtration rate
CVD	Cardiovascular disease
PRS	Polygenic risk score
IQR	Interquartile range
OR	Odds ratio
CI	Confidence interval
PTA	Pure-tone audiometry
