# Supplementary material for: Associations Between Neuropathy, Nephropathy and Hearing Loss in Individuals with Type 2 Diabetes
Source: Biomedicines. 2026 May 20;14(5):1153. doi: 10.3390/biomedicines14051153 (PMC13204839; doi:10.3390/biomedicines14051153)

Supplementary Table S1: DD2 inclusion criteria, definitions, and codes.

**Inclusion criteria up to September 29, 2018**
1. During the entire DD2 enrolment period, the diagnosis of type 2 diabetes in routine clinical practice in Denmark has followed the WHO criteria. Before 2012 this was primarily based on the oral glucose tolerance test (OGTT) or fasting plasma glucose measurements. After 2012 it has primarily been based on glycosylated hemoglobin A (HbA1c) >48 mmol/mol (6.5%). No further diagnostic criteria have been applied in the DD2 project. Moreover, patients are eligible whether they have initiated glucose-lowering therapy at the time of DD2 cohort enrolment or not.<br>Up to September 29, 2018, a "newly diagnosed" type 2 diabetes patient has principally been defined in DD2 as a patient diagnosed later than 1 January 2009, with the recommendation to include only patients with a diabetes duration shorter than 1 year. The median diagnosed diabetes duration at enrolment in the total DD2 cohort is 1.3 years, with an interquartile range [IQR] of 0.3-2.9 years.<br>2. Age ≥18 years<br>3. The patient can give informed consent.
Inclusion criteria after September 29, 2018
After September 29, 2018, the first criterion was changed to "A diagnosis of type 2 diabetes made within the last 24 months"; this is not relevant for our study period. The remaining 2 criteria are unchanged.
Recruitment was done at all Danish outpatient clinics and in approximately 462 general practitioner clinics (1853 clinics exists) at the courtesy of the clinicians at the recruiting sites.

**Data sources**	**Description**
DD2 Biobank	DD2 is a prospective, nationwide population-based cohort of re 2 diabetes individuals, with collection of matching interview da samples for a biobank at baseline. Enrolment started in Novem ongoing.
The Danish Adult Diabetes Registry (DDDA)	The database was established to assess quality in diabetes care 2004. It only covers a subset of the diabetes population.
The Danish National Patient Registry	Covers all inpatient (somatic) hospital contacts since 1977 and f and outpatient hospital contacts in Denmark. Diagnostic inform according to the International Classification of Diseases, Tenth from 1994 onwards. We retrieved information from 1994 and o
The Danish National Prescription Registry (DNHSP)	Cover all redeemed prescriptions at Danish pharmacies since 1
Civil Registration System	All citizens of Denmark are registered in this system by a unique Civi number linked to registrations of birth, address, marital status, kinshi others. The Civil Personal Register number is used as linkage to other
National Lab Database (Laboratoriedatabasen)	The National Lab Database is comprised of detailed informatio tests carried out at Denmark's larger biochemical and immuno started in 2008 and complete coverage of the different regions i from 2010.

**Definitions and codes used in this study**		
**Variable**	**Source**	**Definitions and codes**
Biological sex	Civil Registration System	Male or Female. Defined by social security number in registries.
Age at index date	Civil Registration System and DD2	Age when participants received neuropathy questionnaire.<br>Continuous (years).

Weight at enrolment	Neuropathy questionnaire	Self-reported weight as part of neuropathy questionnaire.
Height	Neuropathy questionnaire	Self-reported height as part of neuropathy questionnaire.
Body Mass Index (BMI) at enrolment	Neuropathy questionnaire	BMI was calculated using the standard formula of weight/height (in metres)^2.
Alcohol consumption		Self-reported at enrolment. Units/week.<br>High-risk intake: >14/21 drinks per week for females/males.<br>High-risk alcohol consumption was categorized according to the Danish Health Authority's definitions as more than 21 and 14 drinks weekly for men and women, respectively, in 2010 when the DD2 was initiated.
Smoking status	Neuropathy questionnaire	Information on smoking through neuropathy questionnaire<br>Categories:<br>• Never smoker;<br>• Former smoker;<br>• Current smoker (comprising daily and occasionally).
Previous cardiovascular disease (CVD)	The Danish National Patient Registry	Any registration of primary or secondary inpatient diagnoses or operation codes (regardless of hospitalization) prior to index date, with a look-back period from DD2 enrolment to 1994, of the following:<br>DI21, DI23, DI24, DT822A (ischemic heart disease); DT823 (acute ischemic heart disease with/without complications); DI20 (angina pectoris); DI25 (chronic ischemic heart disease); KFNA, KFNB, KFNC, KFND, KFNE, KFNF, KFNG, KFNH, KFNW, KFLF (coronary bypass or percutaneous coronary intervention); DI500, DI501, DI502, DI503, DI508, DI509, DI110, DI130, DI132, DI420, DI426, DI427, DI428, DI429 (heart failure); DI48 (atrial fibrillation/flutter); DI61 (cerebral bleeding); DI63, DI64, DI65, DI66 (cerebrovascular infarct); DG45 (transient cerebrovascular disease); DI672, DI678, DI679 (unspecified cerebrovascular disease); DI691, DI693, DI694, DI698 (previous cerebrovascular disease); KAAL10, KAAL11 (cerebral thrombolysis or thromboendarterectomy)<br>DE105, DE115, DE125, DE135, DE145 (diabetes with peripheral vascular complications); DI700, DI701, DI702, DI708, DI709, DI739, DI74, DN280, DK550, DK551, DH340, DH341, DH342 (peripheral/abdominal vascular disease); KNBQ, KNCQ, KNDQ, KNEQ, KNFQ, KNGQ, KNHQ, KPAE, KPAF, KPAH, KPAN, KPAP, KPAQ, KPAW99, KPAU74, KPBE, KPBF, KPBH, KPBN, KPBP, KPBQ, KPBW, KPGH10, KPCE, KPCF, KPCH, KPCN, KPCP, KPCQ, KPCW99, KPCW20, KPCU74, KPCU82, KPCU83, KPCU84, KPGE, KPGF, KPGH, KPGN, KPGP, KPGQ, KPGW99, KPGW20, KPEE, KPEF, KPEH, KPEN, KPEP, KPEQ, KPEW, KPFE, KPFH, KPFN, KPFP, KPFQ, KPFW, KPDU74, KPDU82, KPDU83, KPDU84, KPEU74,

		KPEU82, KPEU83, KPEU84, KPFU74, KPFU82, KPFU83, KPFU84, KPGU74, KPGU83, KPGU84, KPGU99 (vascular surgery).
Metabolic profile		
hsCRP	DD2 biobank	High-sensitivity low-grade inflammation. Measured at DD2 enrolment.
IL-6	DD2 biobank	Interleukin-6. Measured at DD2 enrolment.
TNF-alpha	DD2 biobank	Tumour necrosis factor alpha. Measured at DD2 enrolment.
Medication usage (DNHSP)<br>*For all prescription data the relevant time period is around baseline = DD2 enrollment.*<br>*Thus, the lookback period is 1 year prior to the DD2 enrollment date*		
Antihypertensive drug usage	DNHSP	Up to one year prior to enrolment. Classes and ATC codes:<br>**ACE inhibitors or angiotensin II receptor antagonists:** C09A, C09B, C09C, C09D, C10BX04, C10BX06, C10BX07, C10BX11, C10BX12, C10BX13, C10BX14, C10BX15, C10BX10.<br>**Calcium channel antagonists:** C08, C09BB. C09DB, C09DX01, C09DX03, C09XA53, C09XA54, C07FB, C09BX01, C09BX03, C10BX07, C10BX09, C10BX11, C10BX14.<br>**Low-ceiling diuretics:** C03A, C03B, C03EA, C07D, C09BA, C09DA, C09XA52, C09XA54, C08G, C07B, C09DX01, C09DX03, C09BX03.<br>**Potassium-sparing diuretics:** C03D, C03E.<br>**Beta-blockers:** C07.<br>**Alpha-blockers:** C02CA04, C04CA03.<br>**Central adrenergic inhibition:** C02AC05, C02AB.<br>**Renin inhibitors:** C09XA, C09DX02.
Number of antihypertensive medications	DNHSP	Up to one year prior to enrolment<br>One or more hits in one group counts as one agent: thiazides, potassium-sparing diuretics, beta-blockers, calcium channel antagonists, ACE inhibitors or ATII antagonists, renin inhibitors, alpha-blockers (Doxazosin), central adrenergic inhibitors (Monoxidin, methyldopa)
Lipid-lowering drug usage	DNHSP	Up to one year prior to enrolment.<br>ATC: C10, A10BH51.
GLP-1-analouge or SGLT2 inhibitor usage	DNSHP	GLP1-analogue or SGLT2 inhibitor use (yes/no).<br>ATC:<br>**GLP-1 analogues:** A10BX04, A10BX07, A10BX10, A10BX13, A10BX14, A10BJ, A10AE54, A10AE56.<br>**SGLT2 inhibitors:** A10BX09, A10BX11, A10BX12, A10BD15, A10BD16, ADBD19, A10BD20, ADBD21, A10BK, A10BD23, A10BD24.
Insulin usage	DNHSP	Insulin use (yes/no). Up to one year prior to enrolment.<br>ATC: A10A.
Other glucose-lowering medication	DNSHP	Other glucose-lowering medications (Metformin, **DPP-4 inhibitors, SU and Meglitinides, Thiazolidinediones and alpha-glucosidase inhibitors)** (yes/no) (A10).<br>Classes and ATC codes:<br>**Metformin:** A10BA, A10BD02, A10BD03, A10BD05, A10BD07, A10BD08, A10BD10, A10BD11, A10BD13, A10BD14, A10BD15, A10BD16, A10BD17, A10BD18, A10BD20, A10BD22.<br>**DPP-4 inhibitors:** A10BH, A10BD07, A10BD08, A10BD09, A10BD10, A10BD11, A10BD12, A10BD13, A10BD18, A10BD19, A10BD21, A10BD22.<br>**SU and Meglitinides:** A10BB, A10BD04, A10BD02, A10BD06, A10BD01, A10BC01. A10BX02, A10BX03, A10BX08, A10BD14.<br>**Thiazolidinediones:** A10BG, A10BD03, A10BD04, A10BD05, A10BD06, A10BD09, A10BD12.<br>**Alfa-glucosidase inhibitors:** A10BF, A10BD17.
Number of different antidiabetics including insulin	DNHSP	Up to one year prior to enrolment.<br>One hit or more hits in one group counts as one agent: Sulfonylureas, Meglitinides, Metformin, alpha-glucosidase inhibitors, Glitazones, DD4 inhibitors, GLP-1 analogues, SGLT-2 inhibitors, fast-acting insulin, long-acting insulin.
Complications		

Diabetic polyneuropathy (DPN)	MNSI questionnaire	Defined as a score ≥4.
Nephropathy	Danish laboratory database and DDDA	Defined as microalbuminuria (30–300 mg/g) or macroalbuminuria (>300 mg/g).<br>Collected from DDDA and the National Lab database, using the value closet to index date within -400 days to +400 days.

Supplementary Table S2: DD2 inclusion criteria, definitions, and codes.

**Diagnosis category**	**n**	**ICD-10 codes**
Other diagnoses of hearing loss	348	H91.0, H91.1, H91.2, H91.3, H91.8, H91.8A, H91.9
Noise-induced hearing loss	84	H83.3
Diseases of the inner ear or ear disorders associated with diseases classified elsewhere	13	H83.8, H83.9, H94.0, H94.8
Sensorineural hearing loss or conductive hearing loss	0	H90.0 H90.3, H90.4, H90.5, H90.6, H90.7, H90.8
**Total**	**445**	—

Supplementary Table S3: Age- and sex-adjusted association between neuropathy and hearing loss (HL) stratified by timing of HL diagnosis relative to DD2 enrolment.

Hearing loss timing	***OR***	***95% CI***	***p-value***
Overall			
No hearing loss	*Reference*		
HL >10 years before DD2 enrolment	*1.99*	*1.34–2.96*	*<0.001*
HL 0–10 years before DD2 enrolment	*1.87*	*1.34–2.60*	*<0.001*
HL between DD2 enrolment and index date	*1.73*	*1.05–2.85*	*0.032*
Male			
No hearing loss	*Reference*		
HL >10 years before DD2 enrolment	*1.73*	*1.05–2.84*	*0.031*
HL 0–10 years before DD2 enrolment	*1.38*	*0.90–2.13*	*0.143*
HL between DD2 enrolment and index date	*1.49*	*0.80–2.79*	*0.207*
Female			
No hearing loss	*Reference*		
HL >10 years before DD2 enrolment	*2.61*	*1.34–5.07*	*0.005*
HL 0–10 years before DD2 enrolment	*3.16*	*1.85–5.40*	*<0.001*
HL between DD2 enrolment and index date	*2.26*	*0.97–5.30*	*0.059*

Odds ratios derived from logistic regression models adjusted for age and sex, comparing participants with hearing loss diagnosed at different time points relative to DD2 enrolment with participants without hearing loss.

Supplementary Table S4: Incidence of hearing loss diagnoses after DD2 enrolment according to time between enrolment and index date.

**Time between DD2 enrolment and index date**	**Person-years**	**HL diagnoses (n)**	**Incidence rate (per 1000 person-years)**
**Overall**	9,624	91	9.5
0–1 year	3,406	27	7.9
1–2 years	2,807	32	11.4
2–3 years	2,013	19	9.4
>3 years	1,398	13	9.3

Person-years, number of hearing loss (HL) diagnoses, and incidence rates are shown for participants without a registered HL diagnosis prior to DD2 enrolment. Time categories represent the interval between DD2 enrolment and completion of the neuropathy questionnaire (index date). For example, the category 0–1 year includes participants who were enrolled in DD2 within one year before the questionnaire, whereas >3 years includes participants enrolled more than three years before the questionnaire. Incidence rates are expressed per 1000 person-years.

Supplementary Table S5: Predicted prevalence of hearing loss by sex, age and neuropathy.

**Group**	**Predicted prevalence (%)**	**95% CI**
**Overall**	10.5	9.6–11.4
**Sex**		
Male	12.7	11.5–14.0
Female	7.3	6.1–8.5
**Age (years)**		
40	1.5	1.0–2.0
50	3.2	2.4–3.9
60	6.4	5.5–7.3
70	12.7	11.6–13.8
**Age × Sex**		
Male, 40 years	1.9	1.2–2.5
Female, 40 years	1.0	0.6–1.4
Male, 50 years	3.9	3.0–4.8
Female, 50 years	2.1	1.5–2.7
Male, 60 years	7.9	6.7–9.1
Female, 60 years	4.3	3.4–5.2
Male, 70 years	15.4	13.8–17.0
Female, 70 years	8.7	7.3–10.2
**Sex x Neuropathy**		
Male, no neuropathy	11.9	10.6-13.3
Male, neuropathy	16.9	13.2-20.5
Female, no neuropathy	5.8	4.6-6.9
Female, Neuropathy	13.9	10.3-17.6

Predicted prevalences and 95% confidence intervals were derived from logistic regression models including age and sex. Age-specific estimates represent model-derived predicted probabilities at the specified ages. Estimates for the sex × neuropathy groups represent predicted prevalences of hearing loss according to sex and neuropathy status, illustrating the absolute differences underlying the interaction term reported in the main analysis.

Supplementary Figure S1: Timing of exposure measurements and hearing loss diagnoses relative to the index date.

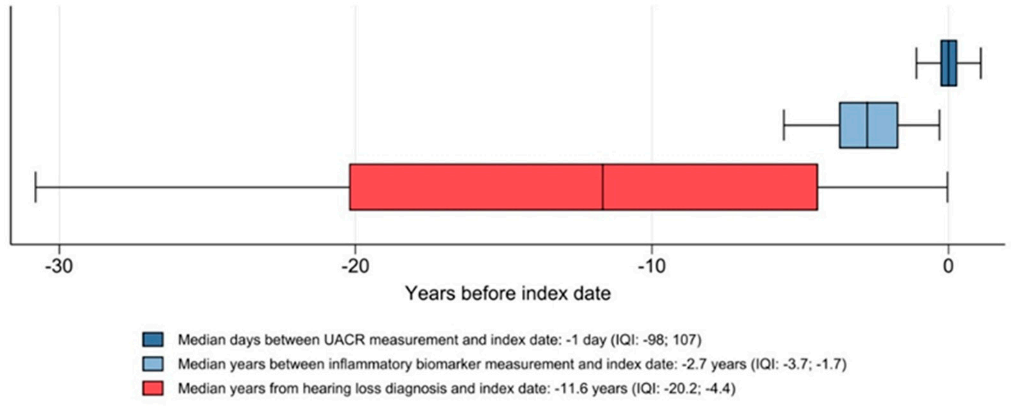

Distribution of time before the index date for: (i) UACR measurements, (ii) inflammatory biomarker measurements, and (iii) the first registry-based hearing loss diagnosis (ICD-10) recorded before the index date.

Supplementary Figure S2: Distribution of observed and imputed values for covariates with missing data.

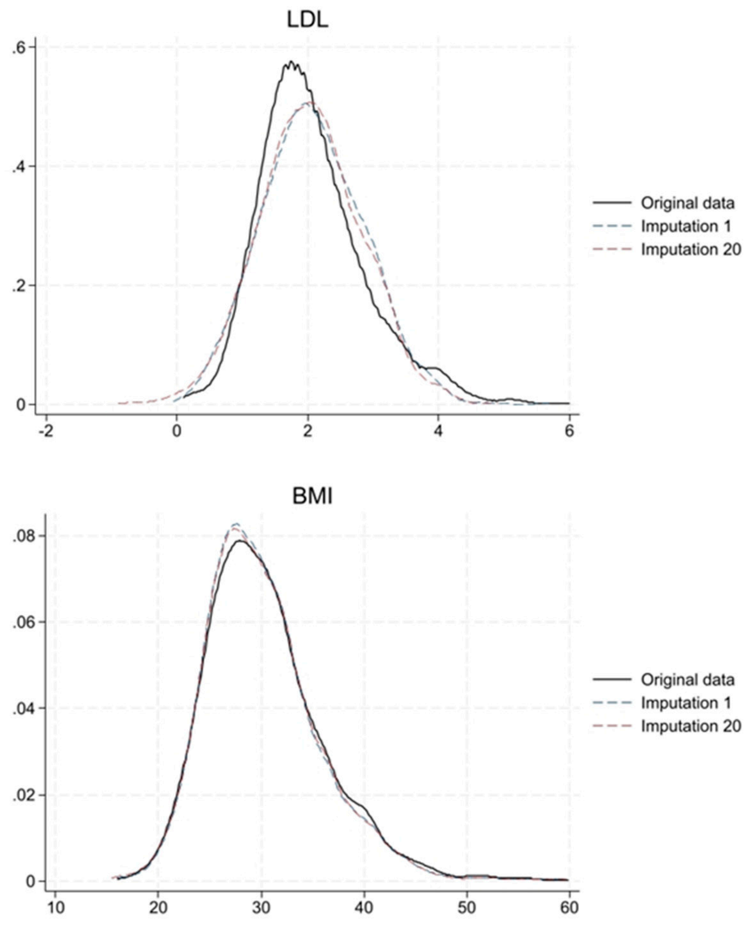

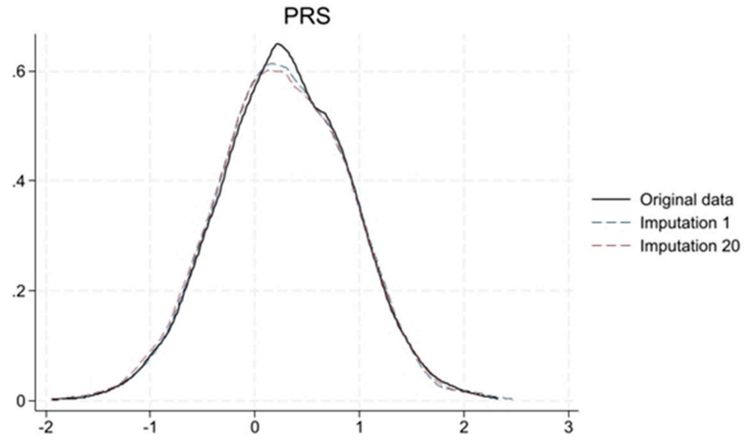

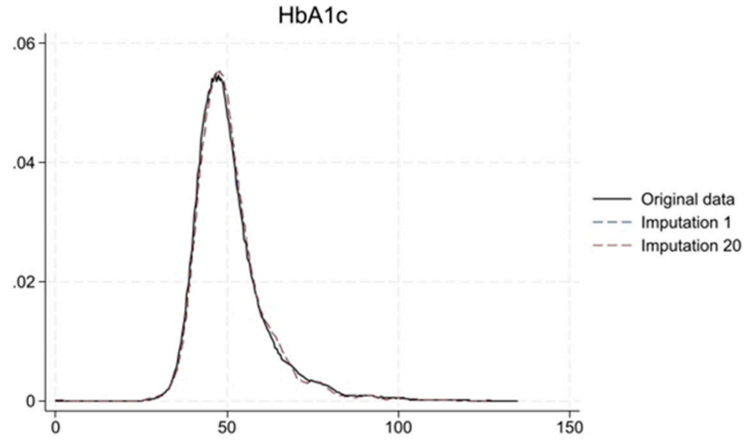

Shown are the original (observed) data and imputed values from the 1st and 20th imputed datasets for (A) LDL cholesterol, body mass index (BMI), (B) polygenic risk score for hearing loss (PRS), (C) body mass index (BMI), and (D) HbA1c. Missing values were imputed using multiple imputation with chained equations including all covariates and exposures, and the outcome.

Supplementary Figure S3: Timing of hearing loss diagnoses in relation to DD2 enrolment and index date.

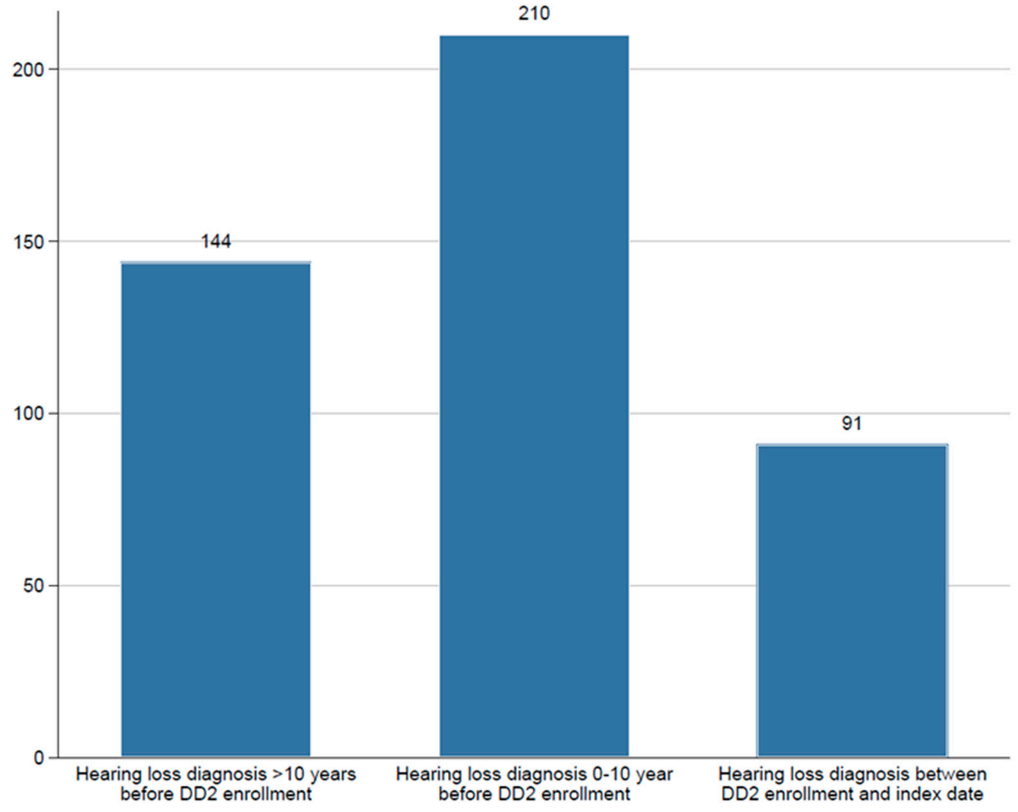

Supplement: Supplementary file 1 [file biomedicines-14-01153-s001.zip › biomedicines-4173602-supplementary.pdf]
